# Reevaluating STEMI: The Utility of the Occlusive Myocardial Infarction Classification to Enhance Management of Acute Coronary Syndromes

**DOI:** 10.1007/s11886-025-02217-8

**Published:** 2025-03-27

**Authors:** Mohammed Ayyad, Maram Albandak, Dhir Gala, Basel Alqeeq, Muath Baniowda, Johann Pally, Joseph Allencherril

**Affiliations:** 1 Department of Internal Medicine, Rutgers New Jersey Medical School, Newark, NJ USA; 2 Department of Internal Medicine, University of Toledo, Toledo, OH USA; 3 Faculty of Medicine, Islamic University of Gaza, Gaza, Palestine; 4 Department of Internal Medicine, University of Missouri-Kansas City, Kansas City, MO USA; 5 University of Illinois Urbana Champaign, Urbana, IL USA; 6 University of Texas Medical Branch at Galveston, Galveston, TX USA

**Keywords:** Occlusive myocardial infarction, Acute coronary syndrome, Electrocardiography, ST-segment elevation, Coronary occlusion

## Abstract

**Background:**

The current classification of acute myocardial infarction (AMI) into ST-segment elevation myocardial infarction (STEMI) and non-ST-segment elevation myocardial infarction (NSTEMI) has limitations in identifying patients with acute coronary occlusion (ACO) who do not exhibit classic ST-elevation. Emerging evidence suggests that a reclassification to "Occlusive Myocardial Infarction" (OMI) may enhance diagnostic accuracy and therapeutic interventions.

**Methods:**

A comprehensive review of the literature was conducted, focusing on the pathophysiology, electrocardiographic (EKG) patterns, and management of ACO. The utility of the OMI paradigm was evaluated against the traditional STEMI/NSTEMI framework, with a particular emphasis on atypical EKG findings and their role in guiding early intervention.

**Results:**

Traditional STEMI criteria fail to identify ACO in approximately 30% of NSTEMI patients, leading to delayed reperfusion and increased mortality. The OMI framework demonstrates improved sensitivity (78.1% vs. 43.6% for STEMI criteria) for detecting ACO by incorporating subtle EKG changes, including hyperacute T-waves, de Winter T-waves, and posterior infarction patterns. OMI-guided management facilitates timely diagnosis and intervention, potentially reducing adverse outcomes. Emerging artificial intelligence (AI) tools further enhance EKG interpretation and clinical decision-making.

**Conclusions:**

Transitioning to the OMI paradigm addresses critical gaps in the STEMI/NSTEMI framework by emphasizing the identification of ACO irrespective of ST-segment elevation. This approach could significantly improve patient outcomes by reducing delays in reperfusion therapy. Future randomized trials are needed to validate the OMI paradigm and optimize its implementation in clinical practice.

## Introduction

The classification of myocardial infarction (MI) has evolved significantly over the past few decades, driven by advances in understanding of coronary pathophysiology. Historically, MI was divided into two major categories based on electrocardiogram (EKG) findings; Q-wave and non-Q-wave infarctions [1]. Q-wave MI was associated with more extensive myocardial damage and believed to be a harbinger of worse clinical outcomes, while non-Q-wave MI, was thought to represent a less severe form [2–4]. However, this simple classification method proved insufficient for guiding therapeutic decisions as understanding of ischemic heart disease progressed. This historical framework was then replaced by the current paradigm, using the nomenclature ST-segment elevation myocardial infarction (STEMI) and non-ST-segment elevation myocardial infarction (NSTEMI) [5–7]. The shift was based on the understanding that ST-segment elevation is a key marker of acute and complete coronary artery occlusion signaling an urgent need for revascularization to restore blood flow and limit myocardial muscle damage [8]. NSTEMI is thought to be typically caused by partial occlusion or reduced blood flow, at times permitting more conservative approach to treatment [9, 10]. This evolution in MI classification has helped define appropriate timing of treatment strategies in patients with acute coronary syndrome (ACS).

The mainstay of treatment is reperfusion of acute coronary occlusion (ACO) which has been shown to significantly reduce mortality and morbidity in patients who present with acute myocardial infarction (AMI) [11]. The only placebo-controlled trials of reperfusion therapy were conducted in the era of thrombolytic therapy, whereby EKGs were classified as having ST-segment Elevation (STE), ST-segment Depression (STD), or neither. A large meta-analysis of these trials revealed that STE was associated with reduced mortality from thrombolytic therapy [11]. However, these trials lacked angiographic confirmation and STE was also poorly characterized. Despite these limitations, STE became a surrogate marker for ACO, leading to the classification of STEMI for cases requiring emergent reperfusion in patients with AMI. No further interventional trials have examined the relationship between STE—or other EKG findings and reperfusion for ACO. Nevertheless, the STEMI vs. NSTEMI paradigm has persisted, and international guidelines continue to use "STEMI" as a surrogate for acute coronary occlusion myocardial infarction. Unfortunately, under this classification, approximately 25–30% of NSTEMI cases involve unrecognized ACO which are only discovered on delayed angiography, typically performed around 24 h after presentation. These patients have approximately double the risk of short and long term mortality rates compared to NSTEMI patients without occlusion [12]. Conversely, 15–35% of catheterization laboratory activations due to perceived STEMI criteria are later found to be false positives, with no identifiable blockages [13, 14]. Another critical consideration is that identifying a coronary occlusion long after the event and subsequent intervention provides no clinical benefit to the patient. Therefore, there is a narrow therapeutic window, making timely diagnosis essential for appropriate intervention, as demonstrated by the OAT trial [15].

Emerging evidence suggests that the current classification may miss patients with ACO who do not exhibit classic STE but still benefit from urgent intervention. Studies have shown that many patients with OMI have subtle or atypical EKG patterns, such as hyperacute T-waves, terminal QRS distortion, and low QRS amplitude, which are not recognized under traditional STEMI criteria [16]. In a recent study, Aslanger et al. reclassified 28% of NSTEMIs as OMI by using structured EKG criteria, identifying a subset of patients with coronary lesions and outcomes similar to those seen in STEMI [17]. Some OMI cases may present with no EKG abnormalities at all, requiring a combination of clinical suspicion, ongoing symptoms, biomarker elevation, echocardiography, or coronary computed tomography angiography (CCTA) to establish the diagnosis [18, 19]. Opposition to the OMI/NOMI classification often references studies suggesting that early angiography in NSTEMI patients does not improve outcomes. However, many of these studies excluded patients with persistent symptoms or did not implement very early intervention [20–24]. For instance, in one large study, patients with persistent symptoms were excluded, and "early" angiography occurred at an average of 16 h from presentation; despite this, patients with high-risk features, such as GRACE (Global Registry of Acute Coronary Events) score greater than 140, did benefit from earlier reperfusion [25]. In studies that did not exclude symptomatic patients and involved truly early intervention, outcomes were better for those randomized to early angiography.

Consequently, international guidelines for NSTEMI recognize the limitations of the current randomized clinical trials and recommend emergent angiography for patients with symptoms highly suspicious for ACS and ongoing instability, even in the absence of definitive EKG or biomarker evidence of AMI [26, 27]. These recommendations reflect the understanding that acute coronary occlusion, rather than specific EKG millimeter criteria, is the underlying pathology warranting emergent reperfusion. This is further supported by a retrospective cohort study by Smith et al., which found that STEMI (-) OMI patients experienced significant delays in diagnosis and reperfusion yet had outcomes similar to those of STEMI ( +) OMI patients [27]. Despite the significantly higher mortality of missed occlusions in NSTEMI patients, no randomized trial has directly addressed this issue due to ethical concerns regarding delayed treatment.

Hence, we propose moving toward the nomenclature "Occlusive Myocardial Infarction" (OMI) to more accurately reflect the pathophysiology of coronary occlusion and better guide therapeutic decisions. Herein we evaluate the potential benefits of transitioning to the OMI classification system. Specifically, we will focus on identifying the EKG patterns associated with OMI and assess whether this approach could improve the timely recognition and management of acute coronary syndromes compared to the current STEMI/NSTEMI distinction. By doing so, we aim to shed light on a paradigm that could potentially enhance clinical outcomes for patients who may benefit from expedited reperfusion therapy and are currently being missed under the STEMI/NSTEMI classification.

## Strengths and Limitations of the Current STEMI/NSTEMI Paradigm

The current STEMI/NSTEMI paradigm has several strengths that have led to an improved patient care in acute myocardial infarction. One of its primary advantages is the ability to differentiate between patients requiring immediate reperfusion therapy and those who may benefit from more conservative or delayed management. This distinction ensures that patients with a STEMI with ACO are rapidly identified and treated with percutaneous coronary intervention (PCI) or fibrinolytic therapy, reducing time to reperfusion, and improving survival outcomes [28–30]. This framework has also facilitated efficient decision-making in emergency settings, enabling clinicians to triage patients based on EKG findings and deliver timely interventions, thereby standardizing care across healthcare systems [5, 31].

Despite its strengths, the STEMI/NSTEMI classification has notable limitations. Its reliance on ST-segment elevation as a marker for ACO excludes many patients with coronary occlusion but without this EKG finding, resulting in misclassification as NSTEMI [32–34]. These patients, who may require urgent revascularization, often receive more conservative management, leading to delays in reperfusion and increased myocardial damage. Additionally, the current STEMI/NSTEMI paradigm may overlook a subset of high-risk patients, highlighting the need for a more refined classification system to better capture these cases.

## The refinement of an OMI Framework

The proposed OMI framework addresses several of the limitations of the current STEMI/NSTEMI paradigm. One of its key advantages is the improved sensitivity and specificity for detecting acute coronary occlusion [35, 36]. By focusing on the presence of total coronary obstruction rather than the strict adherence to ST-segment elevation, the OMI classification can capture a broader spectrum of patients who might otherwise be misclassified as NSTEMI. This approach is especially beneficial in recognizing high-risk patients who present with non-traditional or subtle EKG findings such as isolated ST-segment depressions, deep T-wave inversions, or hyperacute T-waves which may still signify significant coronary occlusion [37].

Emerging evidence suggests that the OMI framework could lead to better patient outcomes. Studies have demonstrated that patients with acute coronary occlusion who do not exhibit ST-segment elevation still face substantial risks of myocardial damage if timely intervention is delayed. Early recognition and intervention in these patients, made possible by the OMI, could reduce reperfusion delays and consequently improve clinical outcomes [38].

## EKG Patterns Suggestive of OMI

Acute coronary syndrome is divided into two main categories: STEMI and non-ST segment elevation acute coronary syndrome (NSTEACS), as determined by initial 12-lead EKG. Current guidelines recommend early reperfusion therapy for STEMI patients as a class I indication [5]. In contrast, NSTEACS encompasses a range of conditions, including unstable angina (without cardiomyocyte damage) and NSTEMI (with cardiomyocyte necrosis). Management of NSTEACS relies on risk stratification, favoring an early invasive approach for high-risk patients, particularly those with elevated cardiac necrosis biomarkers [27, 39].

While STEMI typically results from acute total or near-total occlusion of a coronary artery, instances of complete occlusion of the culprit artery have also been reported in NSTEACS patients. Large retrospective studies indicate that up to 30% of patients with NSTEACS have a totally occluded culprit artery (OCA) [40–42]. One explanation for the presentation of OCA as NSTEACS rather than STEMI is the limited sensitivity of the EKG in detecting acute ischemia or infarction, particularly in the posterior or lateral walls, where the left circumflex artery is often implicated as the culprit [42]. Furthermore, large meta-analyses of non-STEMI patients have revealed that approximately 25% exhibit a completely occluded artery, while 34% show a Thrombolysis in Myocardial Infarction (TIMI) flow grade of 0 to 1 [12, 34]. Notably, these patients experience nearly double the mortality rate compared to non-STEMI patients without OMI, despite being younger and having fewer comorbid conditions [34].

A report addressing the diagnosis of STEMI in the emergency department highlighted the difficulties associated with interpreting EKGs, particularly in cases of borderline ST elevation. It emphasized the need to distinguish STEMI from other conditions such as early repolarization, pericarditis, left bundle branch block, and old myocardial infarction characterized by persistent ST elevation [43]. Additionally, dichotomizing EKGs based solely on the presence or absence of ST elevation limits the ability of STEMI criteria to differentiate between various types of ST elevation. This approach also fails to recognize ST elevation that may be secondary to an abnormal QRS complex combined with underlying primary ischemic elevation [34].

### Atypical Electrocardiographic Patterns in Acute Coronary Occlusion

Within the STEMI framework, cardiologists and emergency physicians have identified occlusion and reperfusion patterns that do not conform to traditional STEMI classifications [44]. These include Wellens' syndrome, characterized by T-wave inversion associated with a risk of reocclusion; de Winter T waves, indicative of left anterior descending (LAD) artery occlusion; the "South African flag sign," which helps identify first diagonal artery occlusion; and the Aslanger pattern, which represents inferior occlusion with concurrent high-grade stenosis. Additionally, ischemic ST depression, particularly in leads V1 to V4, can signal posterior occlusion myocardial infarction and aid in distinguishing it from subendocardial ischemia [34]. Table 1 showcases Essential EKG patterns in the OMI paradigm in more details.

**Table 1:** Essential EKG patterns in the OMI paradigm, detailing characteristic findings, implicated coronary occlusions, and recommended clinical actions

	EKG Pattern	Characteristics	Indicative of	Clinical Implications
*STEMI ( +) OMI*	**ST Elevation**	STE in two contiguous leads	Classical STEMI diagnosis	Urgent reperfusion therapy, high-risk ACS
	**aVR STE with Multi-lead Depression**	STE in aVR with ST depression in other leads	Severe left main or LAD disease or multivessel ischemia	Indicates high-risk patients; urgent intervention required
	**Shark Fin Sign**	Large, wide, and rounded ST-segment elevations that appear as a 'shark fin' pattern	Severe coronary occlusion, often with extensive infarct area	Seen in severe ischemia. Requires urgent reperfusion
	**Transient STEMI**	Brief episodes of ST elevation that resolve spontaneously or with minimal intervention; may lack clear obstructive findings	Transient occlusion or severe spasm without permanent damage	Observation may be needed; recurrent episodes increase risk
	**South African Flag Sign**	STE in noncontiguous leads V2 and aVL with inferior reciprocal ST depression	First diagonal artery occlusion	Requires prompt recognition and intervention
	**Aslanger Pattern**	STE in III with reciprocal STD in I/aVL, with V1 > V2 and ST depression in V5–V6	Inferior OMI with concurrent critical stenosis	Suggests high risk inferior territory occlusion, warranting urgent intervention
	**Smith-Modified Sgarbossa Criteria**	STE concordance or discordant STE/S ratio > 25% in LBBB or ventricular pacing	OMI in cases of LBBB or ventricular-paced rhythm	Sensitive marker for occlusion in complex EKG cases, improving diagnostic accuracy
	**Right Ventricular Infarction**	STE in V1, STE in V1 with ST depression in V2, or ST elevation in III > II; confirmed by ST elevation in right-sided leads (V3R–V6R)	Complicates up to 40% of inferior STEMIs; isolated RV infarction is rare	Patients are preload-sensitive and may develop severe hypotension with nitrates. Treated with fluids; nitrates contraindicated
*STEMI (-) OMI*	**Wellens' Syndrome**	Deep, symmetric T-wave inversion in V2–V3; occurs without STE	LAD artery occlusion, often before infarction	Requires early angiography
	**de Winter Pattern**	Upsloping ST depression at the J point with tall, symmetrical T-waves in precordial leads	Anterior STEMI equivalent, usually LAD occlusion	Urgent reperfusion therapy
	**Posterior MI**	Horizontal ST depression, upright T wave, and tall, wide R wave in V1–V3; R/S ratio > 1 in V2	Occlusion affecting posterior wall, often with inferior or lateral MI	Urgent reperfusion therapy necessitated. Posterior EKG leads may assist in diagnosis
	**Hyperacute T Waves**	Broad, tall T waves that precede STE in early occlusion stages	Early occlusive event in any coronary artery	Prompt identification needed to initiate early intervention
	**Fragmented QRS Complex**	Multiple small notches within QRS complex, typically in ≥ 2 contiguous leads	Marker of ischemic damage, often linked to multivessel disease	Higher risk of ventricular arrhythmias, MACE, and heart failure
	**Primary STD in aVL**	Primary STD in aVL reciprocal to inferior hyperacute T waves	RCA occlusion with inferior ischemia	High specificity for RCA occlusion, useful for differentiating inferior OMI from pericarditis
	**Early Q Waves**	Development of early Q waves, often with hyperacute T waves in the same region	LAD occlusion, often anterior OMI	Early indicator of transmural myocardial infarction, requiring urgent intervention

*STE* ST Segment Elevation, *LAD* Left Anterior Descending artery, *ACS* Acute Coronary Syndrome, *MI* Myocardial Infarction, *aVR* Augmented Vector Right lead on EKG, *MACE* Major Adverse Cardiovascular Event

### Wellen’s Syndrome

Wellen's syndrome is characterized by distinct EKG findings that emergency physicians must recognize, as a significant proportion of patients may progress to anterior wall myocardial infarctions if timely intervention is not initiated. Urgent coronary angiography is warranted for patients with this EKG finding [45]. Quick recognition of these classic T-wave changes can help prevent acute myocardial infarction, particularly in patients without a known history of cardiovascular disease or unmitigated risk factors.

### De Winter T Waves

Similarly, the de Winter EKG pattern is recognized as an anterior STEMI equivalent, primarily characterized by upsloping ST-segment depression at the J point in leads V1–V6, accompanied by tall, symmetrical T waves. This pattern is observed in approximately 2% of patients with subtotal or total occlusion of the proximal LAD artery [46]. Patients presenting with this pattern are experiencing OMI and require immediate reperfusion therapy [47]. Nikus et al. later reported a case of acute myocardial infarction resulting from sub-occlusion of the left circumflex artery, which presented with a similar EKG pattern of upsloping ST-segment depression [48]. It has been noted that patients exhibiting this EKG pattern may progress to typical ST elevation myocardial infarction [49]. A consensus document advised that evidence of this pattern in a patient with suggestive ongoing symptoms and without tachycardia should prompt urgent reperfusion therapy via PCI [49]. The findings discussed above suggest a correlation between upsloping ST-segment depression and myocardial ischemia, but it is essential to recognize that this association is valid only within the appropriate clinical context [50].

### ST Depression in Anterior Precordial Leads as an Indicator of Posterior AMI

Acute posterior wall MI accounts for approximately 15% to 21% of all acute MIs, predominantly occurring alongside acute infarction of the inferior or lateral walls of the left ventricle. Detecting acute posterior wall MI presents challenges, as the standard 12-lead EKG inadequately visualizes the posterior wall of the left ventricle [51]. Furthermore, the electrocardiographic criteria indicative of acute posterior wall MI are not well-known among practitioners. The lack of evident ST elevation often leads to missed diagnoses, making posterior wall MI one of the most commonly overlooked patterns of acute infarction in electrocardiographic assessments [52].

Several electrocardiographic findings indicative of acute posterior wall MI can be observed in the 12-lead EKG, particularly in leads V1, V2, or V3. These findings include: 1) horizontal ST segment depression, 2) a tall, upright T wave, 3) a tall, wide R wave, and 4) an R/S wave ratio greater than 1.0 in lead V2 [53]. Additionally, the presence of both horizontal ST segment depression and an upright T wave enhances the diagnostic accuracy of these electrocardiographic findings [53]. Therefore, adopting the OMI paradigm, which focuses on the presence or absence of acute coronary occlusion rather than solely relying on ST elevation on EKG, could enhance the detection of cases requiring interventions for acute coronary occlusion, even when ST elevation is absent.

### ST Elevation in AVR Lead (LMCA/Proximal LAD Occlusion or Multivessel Disease)

The American Heart Association guidelines maintain that STE must be present in at least two contiguous leads for a STEMI diagnosis. However, the 2013 update introduced the recognition of isolated STE in the augmented vector right (aVR) lead, accompanied by multi-lead ST depression, as a potential indicator of STEMI due to left main coronary artery (LMCA) or proximal LAD occlusion [5]. Previous studies have identified two primary clinical scenarios associated with ST elevation in lead aVR. The first scenario involves total occlusion of the LMCA or the proximal LAD, where STE in lead aVR indicates ischemia in the basal interventricular septum and is not considered reciprocal [54]. Timely reperfusion in these cases is essential for a favorable outcome. The second scenario occurs in patients with severe multivessel disease without acute occlusion, resulting in subendocardial ischemia that produces ST segment depression in the inferolateral leads alongside reciprocal elevation in aVR [55–57]. This group is considered high-risk within the acute coronary syndrome population. However, it is important to note that a study found only 10% of patients with ST segment elevation in lead aVR have an acutely occluded coronary artery [53].

### Fragmented QRS Complexes and Hyperacute T Waves

Other EKG findings indicative of coronary occlusion that do not present as ST elevation include hyperacute T waves and fragmented QRS complexes. Fragmented QRS complexes are multiple small notches within QRS complex, typically in ≥ 2 contiguous leads. They are significantly associated with both in-hospital and long-term mortality, as well as major adverse cardiovascular events (MACE) in patients with acute myocardial infarction. They are also positively correlated with a higher incidence of triple vessel coronary artery lesions [58]. Additionally, they are linked to an increased risk of ventricular arrhythmias and heart failure, potentially serving as a marker for mortality and MACE risk [58]. Hyperacute T waves typically appear shortly after the onset of coronary occlusion and transmural infarction, evolving rapidly into STE [59].

### Shark Fin Sign

Also referred to as triangular QRS-ST-T waveform, lambda-wave, or giant R waves. It appears as a giant R wave with an amplitude of > 1 mV and a QRS complex fused with the ST-segment and T-wave. This pattern is linked to massive myocardial ischemia, particularly resulting from LAD or LMCA occlusion [60, 61]. Due to its rarity, occurring in only 1.7% of STEMI cases, the shark fin pattern demands high clinical suspicion and prompt intervention. It is also associated with a significant risk of atrial and ventricular fibrillation, contributing to higher in-hospital mortality rates [62].

### South African-Flag Pattern

This sign signifies occlusion of the first diagonal branch (D1) of LAD. It manifests as ST-elevation in leads I, aVL and V2 along with ST-depression in lead III and inferior leads resembling the south African flag shape [63, 64].

### Left Bundle Branch Block (LBBB) with Smith-Modified Sgarbossa Criteria

A diagnosis of ACO can be difficult to make in patients with LBBB due to appropriate discordance in which an abnormal depolarization (i.e., ST-segment and T-wave deviation) is followed by an abnormal repolarization which are not -necessarily- secondary to coronary artery occlusion. However, serial ECG can be helpful in such situation while taking into account Smith-Modified Sgarbossa Criteria which have been developed to improve the sensitivity and specificity of ECG in the detection of ACO in LBBB [65]. They diagnose ACO in patients LBBB depending on the presence of one of the following: (1) concordant ST-elevation > 1 mm in one lead or more (5 points), (2) concordant ST-depression > 1 mm in one lead or more of V1-V3 (3 points) or (3) Proportionally excessive discordant ST-elevation in any lead with ≥ 1 mm STE as defined by ≥ 25% of the depth of the preceding S-wave (2 points) [60]. Those criteria have relatively low sensitivity but are highly specific (with 90% specificity for a score of 3 or more); making them a very useful tool when encountering patients with LBBB in appropriate clinical scenarios with high suspicion for ACO.

### Transient STEMI

While presenting with typical symptoms for ACO and having ST-changes typical for STEMI, patients with this clinical entity have spontaneous resolution of symptoms and electrocardiographic changes prior to reperfusion therapy and represent up to 24% of STEMI cases [66]. In a study by Meisel et al., patients with transient STEMI were found to have resolution of symptoms and electrocardiographic changes in 1.2 ± 0.8 h after presentation. While the exact approach for managing this condition is not yet standardized, some evidence suggest that early medical treatment followed by invasive reperfusion therapy is recommended [67]. Fortunately, patients with transient STEMI generally exhibit a more favorable prognosis compared to those with persistent STEMI, potentially due to factors such as spontaneous recanalization of the infarct-related artery or transient epicardial coronary artery spasm [66].

### ST-Segment Depression in Lead aVL

Differentiating inferior STEMI from pericarditis represents a common and significant clinical dilemma for physicians. A retrospective cohort study involving 154 patients with inferior STEMI and 49 patients with pericarditis examined the diagnostic value of ST-depression in lead aVL in distinguishing between these two conditions. The study found that ST-depression in lead aVL is 100% specific for diagnosing ACO due to inferior STEMI over pericarditis [68].

### Old Q waves versus New STEMI

Another conundrum that is faced in clinical practice is the presence of Q waves with concurrent ST-elevation in the anterior leads which can muddy the distinction between acute anterior STEMI and left ventricular (LV) aneurysm [69]. A useful tool was developed by Smith et al. in which the ratio of highest T/QRS amplitudes in leads (V1-V4) is measured. If this ratio is shown to be more than 0.36, a diagnosis of STEMI can be made with 90% accuracy [70].

## Clinical Implications

In the STEMI/NSTEMI framework, relying solely on the standard 12-lead EKG may lead to the omission of patients with ACO, as STEMI criteria alone are a poor surrogate for ACO [71, 72]. One major challenge is the limited sensitivity of 12-lead EKGs in detecting ACO. A systematic review and meta-analysis found the sensitivity of EKG for detecting ACO in STEMI patients was only 43.6%, implying more than half of cases of ACO had no ST-elevation [73].

## Occlusive MI Framework Implementation: Clinician Training and EKG Interpretation

Implementing the OMI paradigm shift requires enhancing clinician education, awareness, and training in EKG interpretation. Educational programs are essential to increase EKG determination accuracy. A systematic review and meta-analysis highlighted that physicians' accuracy in EKG interpretation varies widely, with a median accuracy of 54% pre-training and 67% post-training, leaving substantial room for enhancement [74]. Furthermore, EKG pattern interpretation is largely individual dependent and thus prone to misinterpretation and human error. The American Heart Association emphasizes the importance of ongoing training for physicians and nurses in EKG interpretation [75]. In addition, it is essential to properly educate residents on identifying specific EKG changes that can suggest ACO (including hyperacute T waves, de Winter's sign, or Wellens' syndrome). The American College of Cardiology cautions clinicians to pay attention to EKG changes that are harder for the human eye to detect, which may signal early vessel occlusion and necessitate emergent coronary angiography [71].

McLaren et al. stress the significance of quality improvement programs using standardized feedback and case discussions that will ultimately accelerate ACO identification and treatment without increasing false activation [76]. Furthermore, a new artificial intelligence tool (i.e., Queen of Hearts) has been developed to detect coronary artery occlusion regardless of the presence of ST-elevation with promising performance at 94% specificity and 81% sensitivity [77].

## Changes in Sensitivity and Specificity for MI detection

The effective application of OMI criteria to patients with acute MI has important consequences on both the sensitivity and specificity for detecting MI as well as long-term patient events. Patients who meet OMI criteria, especially those with NSTEMI, but have coronary occlusion that needs to be treated urgently, otherwise portend poor long-term results. Studies have shown that these patients are at similar risk of morbidity and mortality if catheterization is delayed as those with STEMI [35].

The OMI criteria enhance the ability to detect acute coronary occlusions compared to the traditional STEMI criteria. A systematic review and meta-analysis by de Alencar Neto et al. found that while STE alone has a sensitivity of about 43.6% for identifying acute coronary occlusion, the OMI criteria raise this sensitivity to 78.1% without a notable impact on specificity. This improved sensitivity decreases false negative data and is crucial for promptly identifying patients who need immediate reperfusion therapy [73]. Furthermore, the OMI paradigm enhances the detection of coronary occlusion in patients who do not show the typical ST elevation. For example, significant ST-segment depression in leads V1-V4 has a high specificity of 97% for identifying OMI, even without ST elevation. This approach helps reduce the number of cases that might go undetected by traditional STEMI/NSTEMI criteria [38].

The OMI criteria are designed to enable earlier and more accurate diagnosis of ACO. Clinicians can recognize coronary occlusion earlier by detecting minor changes in EKG as well as utilizing sophisticated diagnostics such as machine learning models that improve patient health prospects through faster initiation of treatment [78, 79].

## Impact of the OMI Paradigm Implementation on Management and Treatment

The OMI paradigm simply asserts all patients with ACO need immediate activation/intervention, irrespective of classic ST-segment elevation. The idea of this approach is that patients with high-risk EKG findings (subtle ST-segment changes or hyperacute T waves) be sent for early coronary angiography, to receive timely and appropriate reperfusion therapy [35]. Thus, the implementation of the OMI paradigm requires updating clinical protocols and guidelines. This requires robust revision of the recommendations provided by the American College of Cardiology and other societies to include OMI-specific criteria that would facilitate rapid invasive revascularization for patients with acute coronary occlusions as is already recommended in those with STEMI.

Even with the development of high-sensitivity troponin tests, EKG interpretation remains essential. Proper EKG interpretation, particularly noticing subtle changes that suggest OMI, is key to making a timely diagnosis and initiating treatment [80].

Patients with acute coronary occlusion who fail to meet conventional STEMI criteria yet are identified by OMI criteria share a similar adverse outcome as their “STEMI'' cohorts when revascularization is delayed. It may be required to reduce mortality and morbidity because they could ensure that no ACO patient is admitted to a hospital without being treated with reperfusion therapy [80]. All that being said, the lack of randomized clinical trial validation of the OMI paradigm in comparison to the STEMI paradigm represents a significant limitation for the applicability of such paradigm.

## Future Directions

The literature review uncovers important details and pitfalls in the STEMI/NSTEMI approach to treating ACO. Although traditional STEMI/NSTEMI criteria are widely used, they often fail to identify patients with ACO who lack the classic ST-elevation pattern. This highlights the potential value of detecting occlusions through more subtle EKG changes, as captured by the OMI rule, which may be missed by traditional criteria in real-world clinical practice [81].

Existing evidence suggests that dependence on the STEMI/NSTEMI dichotomy alone to characterize ACS will fail to diagnose and treat many patients in an expedited fashion, resulting in worse outcomes associated with delayed treatment.

The OMI criteria could aid clinicians to recognize the non-classical EKG patterns which are often under-recognized by current clinical guidelines. Incorporating these subtle EKG findings into daily practice may result in more accurate and timely anterior wall AMI diagnoses [82]. A promising advancement in this area is the use of AI to augment clinical decision-making. Software platforms like PowerfulMD, Queen of Hearts, and similar AI-driven tools are increasingly capable of detecting subtle EKG patterns that may be challenging for even the most experienced clinicians to recognize. These AI systems can assist in identifying non-classical signs of ACO, potentially improving early diagnosis. As AI technology evolves, it holds the potential to significantly enhance diagnostic accuracy and streamline clinical workflows, ensuring more timely and precise interventions for patients at risk [35].

There is considerable potential for further research and education on non-classical EKG patterns suggesting the presence of ACO. Patients recognized through OMI criteria, especially those with NSTEMI-ACO, appear to be at high risk of adverse events similar to STEMI and should not experience delays in reperfusion. Research has shown that this group faces increased odds of mortality and delays in catheterization procedures. Early recognition and intervention according to the OMI criteria could substantially improve long-term patient outcomes by guaranteeing timely reperfusion therapy [12, 35]. Randomized controlled trials comparing the STEMI and OMI paradigms are mandatory to validate the benefits of the latter approach for earlier recognition and management of ACO.

## Key References

- Kola M, Shuka N, Meyers HP, Zaimi (Petrela) E, Smith SW. OMI/NOMI: Time for a New Classification of Acute Myocardial Infarction. J Clin Med. 2024;13(17):5201. 10.3390/jcm13175201.
  - ○ This article proposes a paradigm shift from STEMI/NSTEMI classification to Occlusion MI (OMI) and Non-Occlusion MI (NOMI), addressing the limitations of traditional EKG interpretations in acute coronary syndrome.
- McLaren J, De Alencar JN, Aslanger EK, Meyers HP, Smith SW. From ST-Segment Elevation MI to Occlusion MI. JACC Adv. 2024;3(11):101,314. 10.1016/j.jacadv.2024.101314.
  - ○ This paper highlights the clinical implications of misclassifying occlusive myocardial infarctions under the STEMI paradigm, advocating for the adoption of OMI to improve diagnostic accuracy and patient outcomes.
- de Alencar Neto JN, Scheffer MK, Correia BP, Franchini KG, Felicioni SP, De Marchi MFN. Systematic review and meta-analysis of diagnostic test accuracy of ST-segment elevation for acute coronary occlusion. Int J Cardiol. 2024; 402:131,889. 10.1016/j.ijcard.2024.131889.
  - ○ This meta-analysis comprehensively evaluates the diagnostic accuracy of ST-segment elevation in identifying acute coronary occlusion, underscoring the need for more refined criteria to enhance clinical decision-making.

## Data Availability

No datasets were generated or analysed during the current study.

## Abbreviations

ACO: Acute Coronary Occlusion
AI: Artificial Intelligence
AMI: Acute Myocardial Infarction
ACS: Acute Coronary Syndrome
AVR: Augmented Vector Right (EKG lead)
CCTA: Coronary Computed Tomography Angiography
D1: First Diagonal Branch of the Left Anterior Descending Artery
EKG: Electrocardiogram
GRACE: Global Registry of Acute Coronary Events
LAD: Left Anterior Descending Artery
LMCA: Left Main Coronary Artery
LV: Left Ventricle
LBBB: Left Bundle Branch Block
MACE: Major Adverse Cardiovascular Events
MI: Myocardial Infarction
NSTEMI: Non-ST-Segment Elevation Myocardial Infarction
NSTEACS: Non-ST-Segment Elevation Acute Coronary Syndrome
OMI: Occlusive Myocardial Infarction
OCA: Occluded Culprit Artery
PCI: Percutaneous Coronary Intervention
QRS: A specific wave pattern in an EKG
REF: Reference (used for studies)
STE: ST-Segment Elevation
STD: ST-Segment Depression
STEMI: ST-Segment Elevation Myocardial Infarction
TTE: Transthoracic Echocardiogram
TIMI: Thrombolysis in Myocardial Infarction
